# Adsorption Characteristics of Iron on Different Layered Loess Soils

**DOI:** 10.3390/ijerph192416653

**Published:** 2022-12-11

**Authors:** Li He, Yonghui Huang, Zhigang Xie, Wei Guan, Yao Zeng

**Affiliations:** 1 College of Resources and Environment, Zunyi Normal University, Zunyi 563006, China; 2 Chengdu Drainage Co., Ltd., Chengdu 610000, China; 3 Chongqing Key Laboratory of Environmental Materials & Remediation Technologies, Chongqing University of Arts and Sciences, Chongqing 402171, China; 4 Environment Monitoring Station of Dadukou District, Chongqing 400084, China

**Keywords:** soil adsorption, mine wastewater, loess soil, kinetics

## Abstract

In view of the problem of Fe^3+^ pollution in an iron sulfur mine, different layers of loess soil in the Bijie area were used for adsorption to alleviate the mine wastewater pollution by natural treatment. The effects of the initial concentration of Fe^3+^, adsorption time and pH value on the adsorption performance of top, core and subsoil layers of loess soils were studied by the oscillatory equilibrium method, and the adsorption mechanism of these three soils was analyzed through a kinetic adsorption experiment and infrared spectroscopy. The results showed that the adsorption capacity of Fe^3+^ was improved by increasing the initial concentration and reaction time, but the adsorption rate of the adsorption capacity of Fe^3+^ was reduced. The adsorption rate of Fe^3+^ in the subsoil layer was faster than that in the other two layers. The higher the pH, the higher the adsorption capacity. After the pH was higher than 3.06, it had little effect on the adsorption capacity, but the adsorption rate increased. The first-order kinetic equation, second-order kinetic equation and Elovich equation were suitable for iron adsorption kinetics of three soils. The fitting correlation coefficient of the second-order kinetic equation was close to one, indicating the main role of chemical adsorption. The adsorption rate constant of the subsoil layer was about two times and three times that of the core soil layer and the topsoil layer. The Langmuir model can better fit the isothermal adsorption process. The results of infrared spectroscopy of soil showed that the content of soil organic matter played an important role in the adsorption capacity of Fe^3+^. The subsoil layer had a higher concentration of organic matter and more abundant functional groups, so the adsorption capacity of Fe^3+^ was the highest. The results could provide a theoretical basis for the removal of iron in acid mine wastewater.

## 1. Introduction

China is rich in pyrite resources, the main components of which are marcasite, pyrite and pyrrhotite. Various forms of Fe, under the action of microorganisms (ferrobacillus ferrooxidant) and O_2_, generated acid mine wastewater during the mining process. According to some report, the concentration of Fe^3+^ in acidic mine wastewater was more than 1000 mg·L^−1^, and the high concentration of iron ion and its complex made the mine wastewater reddish brown in color, seriously threatening the water quality safety of surface water and underground water [1]. A large amount of acid mine wastewater flowed into the nearby farmland to form coal rust fields, resulting in a large area of soil pollution and affecting the growth of rice and other food crops [2]. When the concentration of iron in water is 0.1–0.3 mg/L, the color, smell and taste of water will be affected [3]. Thus, the limit value of Fe in <Standards for drinking water quality> (GB5749-2022) is 0.3 mg/L. If the iron in the sewage is too high, it will inhibit the activity of microorganisms and even cause microbial death. When the iron in the wastewater reaches 5 mg/L, the sludge precipitation and fermentation in the sedimentation and digestion tank will also be affected.

Traditional acid mine wastewater treatment methods include active treatment based on alkaline neutralization and passive treatment based on reduction treatment using sulfate reducing bacteria (SRB) [4,5]. Although the active treatment method was simple to operate and achieve, simultaneously adjusting pH and precipitating iron ions, the cost of adding large dosage of alkaline reagents was high, and the chemical sediment needed to be removed regularly. The passive treatment method using SRB bacteria can achieve the reduction of the sulfate ions; on the one hand, the acidity was reduced, and on the other hand, FeS precipitation was generated through the reaction between S^2−^ and iron ions to remove iron ions. However, the sewage treatment process required the construction of sewage ponds, and the treatment cycle was long, which was not suitable for small-scale wastewater treatment.

Soil as a natural adsorbent was often used in the adsorption studies of heavy metal ions such as plumbum [6,7,8], cadmium [9], arsenic [10], copper [11], chromium [12] and zinc [13]. Although there was no published paper on soil adsorption of iron, He et al. [14] used red clay and bentonite to treat metal in mine wastewater, including iron. According to the preliminary investigation, there are a large number of abandoned small coal mines in Bijie, Guizhou Province, and the mine wastewater and coal rust fields are seriously polluted. There is a very common phenomenon in nearby villages that the mine wastewater is filtered through the soil and used for vegetable irrigation. According to ICP measurement, Fe^3+^ filtered by soil can meet the standard of <The reuse of urban recycling water-Quality of farmland irrigation water> (GB 20922-2007). However, the treatment conditions and mechanism are still unclear.

However, there were few studies on the adsorption of iron ions. Soil can be divided into the top, core and subsoil layers, and each layer of soil had different physical and chemical properties such as organic matter content and particle size composition, which showed different adsorption characteristics for metal ions. In this research, taking the top, core and subsoil layers of yellow soil in Bijie area as samples, the adsorption characteristics of ferric iron for different layers of soil were studied in the laboratory. Finally, the infrared spectrum characterization of the soils before and after adsorption were carried out to provide a theoretical basis for the removal of iron in acid mine wastewater.

## 2. Materials and Methods

### 2.1. Reagents

Ammonium ferrous sulfate ((NH_4_)_2_Fe(SO_4_)_2_·6H_2_O)), sulfuric acid (H_2_SO_4_), hydrochloric acid (HCl), hydroxylamine hydrochloride (HONH_3_Cl), ammonium acetate (CH_3_COONH_4_), acetic acid (CH_3_COOH) and phenanthroline (C_12_H_8_N_2_·H_2_O) were provided with Aladdin Co., Ltd. (Shanghai, China). Besides, all the reagents were analytically pure and dissolved using the deionized water. Taking the top, core and subsoil layers of yellow soil in Bijie area as samples, the physical and chemical properties of each soil were presented in Table 1. The soil sample was dried in the dark, the sundries in soil sample was removed, and the soil sample was grounded before analyzing these samples.

### 2.2. Characterization and Analysis

The soil organic matter was determined using colorimetry through hydration thermal oxidation of potassium dichromate. The pH of the soil was determined by soil and water at the ratio of 2.5:1, and the pH of the soil was measured by A FE28 pH meter (Meter, New York, NY, USA). The concentrations of cation exchange capacity (CEC) in soil were measured by flame photometry. Soil particle distribution was measured by a malvern laser particle size analyzer (Mastersizer 2000, Malvern, UK). The soil before and after adsorption was characterized by FTIR spectrometer (IRPrestige-21, Shimadzu, Tokyo, Japan). The concentrations of iron and manganese in soil were determined by atomic absorption spectrophotometry (AA-80, Shimadzu, Japan), and the concentration of iron ions in wastewater was determined by phenanthroline spectrophotometry (UV-5900PC, Shanghai Yuanxi, Shanghai, China).

### 2.3. Adsorption Experiments

We weighed 4 g soil and put it into 100 mL centrifuge tubes, then added 70 mL of the ammonium ferrous sulfate solution into the reactor (the concentrations for the solution were 50 mg·L^−1^, 100 mg·L^−1^, 200 mg·L^−1^, 300 mg·L^−1^ and 400 mg·L^−1^). After a 20 min reaction in the oscillator and being centrifuged for 10 min, a 1 mL water sample was taken into a 50 mL conical flask. Then, all the centrifuges were shaken well and reacted in the oscillator for 40 min, then centrifuged for 10 min. Next, a 1 mL water sample was taken in a 50 mL conical flask. We repeated the operation and a 1 mL water sample was taken for 60 min, 90 min and 180 min, respectively. The influence of initial pH (2.48, 3.06 and 4.02) on adsorption performance of soil was analyzed.

The adsorption capacity of iron in soil was calculated according to the following Equation (1):

(1) $$Q=\frac{(C_{0}-C)\times V}{m}$$

where *Q* was the amount of soil adsorption of heavy metals, mg·g^−1^; *C* was the equilibrium concentration of heavy metals in the solution, mg·L^−1^; *C*_0_ was the initial concentration of heavy metals in the solution, mg·L^−1^; *V* was the volume of liquid at equilibrium, L; *m* was the mass of soil samples, g.

The equilibrium adsorption kinetics model of metal ions on soil surface was most widely used, such as the first-order reaction adsorption equation, second-order adsorption equation and Elovich equation, which were shown, as following, respectively [15]:

ln(*q_m_* − *q*) = ln*q_m_* − *kt* (2)

*t*/*q* = 1/(*kq_m_*^2^) + *t*/*q_m_* (3)

*q* = *b* + *k*ln*t* (4)

where *t* was reaction time, minute; *q* represented the adsorption amount of Fe^3+^ (mg·g^−1^) when the reaction time was *t*; *q_m_* represented the maximum saturated adsorption amount of Fe^3+^ (mg·g^−1^); *b* and *k* were the constants.

Langmuir and Freundlich isotherm adsorption models are selected for calculation. The formulas are shown in (5) and (6).

*C_e_*/*q_e_* = *C_e_*/*q_m_* + 1/(*b* × *q_m_*) (5)

ln*q_e_* = ln*K* + (1/*n*) × ln*C_e_* (6)

where *q_e_* is the equilibrium adsorption quantity (mg/g), *C_e_* is the equilibrium concentration (mg/L); *b* and *K* is the model fitting constant; 1/*n* is the anisotropy index.

## 3. Results and Discussion

### 3.1. Effect of Initial Concentration of Fe^3+^ on Soil Adsorption Capacity

**Figure 1:**
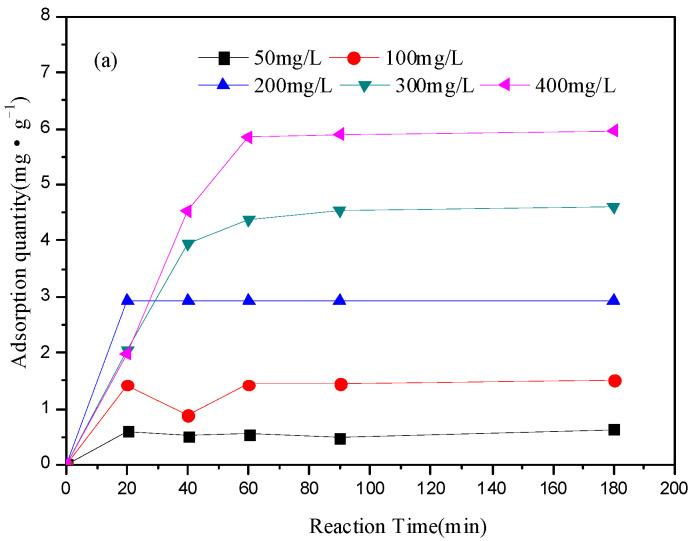

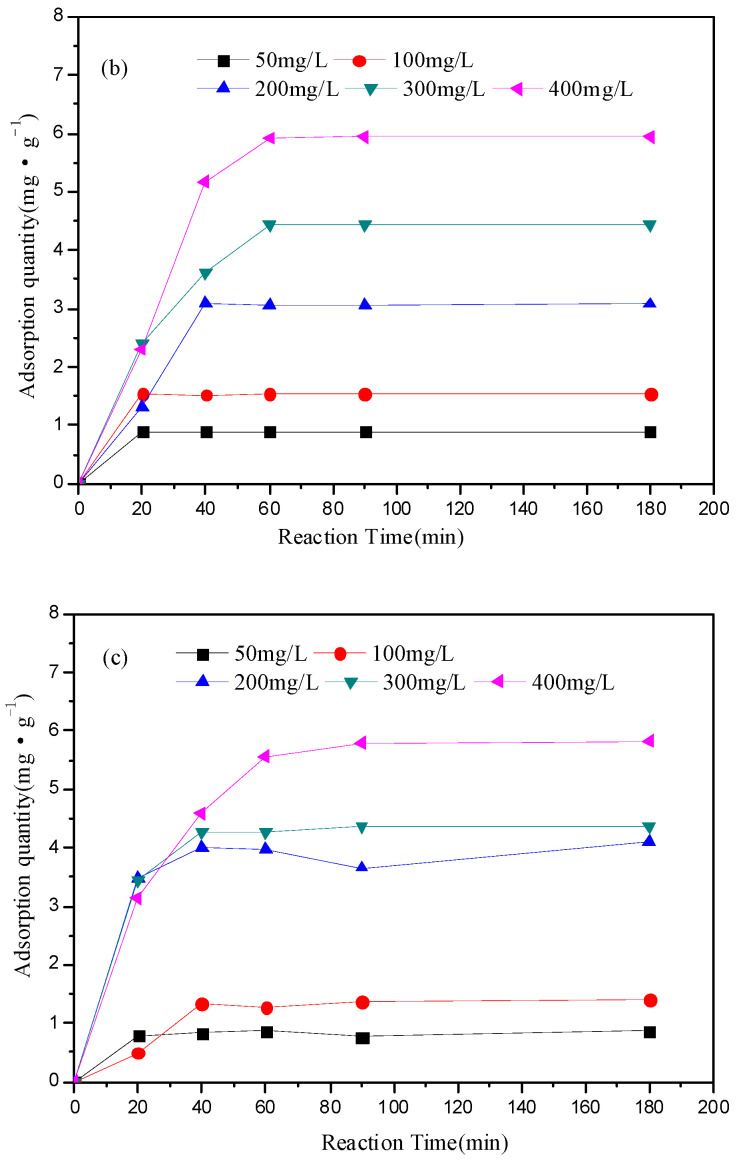
Effect of initial concentration of Fe^3+^ on adsorption capacity of the three layers of soil: (**a**) top layer of loess soils; (**b**) core layer of loess soils and (**c**) subsoil layer of loess soils. (4 mg of soil, pH = 4.20, 25 °C).

The effect of the initial concentrations of Fe^3+^ (50 mg·L^−1^, 100 mg·L^−1^, 200 mg·L^−1^, 300 mg·L^−1^ and 400 mg·L^−1^) on the adsorption capacity of the three layers of soil was shown in Figure 1. The adsorption capacity of each layer of soil was increased gradually with the increase of the initial concentration of Fe^3+^ and reaction time; oppositely, the adsorption rate was slower. For the top layer, the adsorption equilibrium was reached at 20 min when the initial concentration of Fe^3+^ was lower than 200 mg·L^−1^. However, the adsorption equilibrium was reached at about 60 min when the concentration of the initial concentration of Fe^3+^ was higher than 200 mg·L^−1^. For the core layer, the adsorption equilibrium was reached at 20 min when the initial concentration of Fe^3+^ was lower than 100 mg·L^−1^. The adsorption equilibrium was reached at 40 min when the initial concentration of Fe^3+^ was 200 mg·L^−1^. However, the adsorption equilibrium was reached at about 60 min when the concentration of the initial concentration of Fe^3+^ was higher than 300 mg·L^−1^. For the subsoil layer, the adsorption equilibrium was reached at 40 min when the initial concentration of Fe^3+^ was lower than 300 mg·L^−1^. However, the adsorption equilibrium was reached at about 60 min when the concentration the initial concentration of Fe^3+^ was higher than 400 mg·L^−1^. The maximum adsorption capacity of the three soils was about 6 mg·g^−1^ when the initial concentration of Fe^3+^ was 400 mg·L^−1^. In general, the subsoil layer had a faster adsorption rate for the high concentration of Fe^3+^ than the other two soils. Moreover, the adsorption capacity had been increasing with the increase of the initial concentration of Fe^3+^, indicating that the adsorption site for iron in each layer was much higher than 400 mg·L^−1^.

### 3.2. Effect of Initial pH on Soil Adsorption Capacity

**Figure 2:**
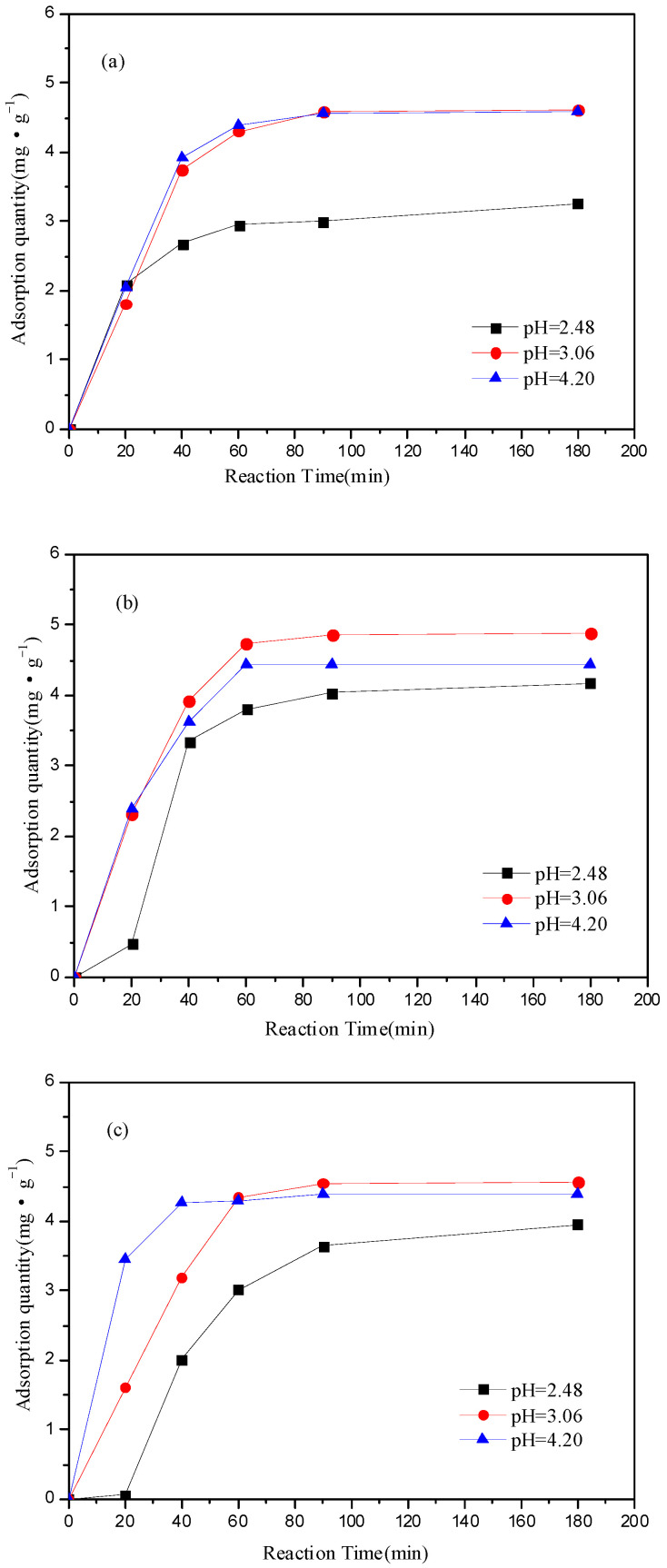
Effect of initial pH (2.48, 3.06 and 4.20) on the adsorption capacity of the three layers of soil: (**a**) top layer of loess soils; (**b**) core layer of loess soils and (**c**) subsoil layer of loess soils. (4 mg of soil, initial concentration of Fe^3+^ was 300 mg·L^−1^, 25 °C).

The effect of the initial pH (2.48, 3.06 and 4.20) on the adsorption capacity of the three layers of soil was shown in Figure 2. In general, the higher pH value, the higher adsorption capacity of the soil. The adsorption capacity was not changed obviously when the pH value was higher than 3.06, but the adsorption rate was affected by the pH value. The adsorption rate of the subsoil layer at a pH value of 4.20 was faster than that at a pH value of 3.06, but it showed little effect on the adsorption rate of top layer and core layer of soil. The adsorption equilibrium was reached at about 60 min for both the top layer and core layer when the pH value was 4.20, and the maximum adsorption capacity was 4.5 mg·g^−1^, whereas the adsorption equilibrium was reached at about 40 min for the subsoil layer, and the maximum adsorption capacity was 4.38 mg·g^−1^. The results showed that the maximum adsorption capacity of the three soils were very similar, but the adsorption rate of the subsoil layer was obviously faster than that of the other two soils, which was consistent with the result in Figure 1.

Surface charge and the functional groups on the soil surface and Fe^3+^ form would be influenced by the pH value, which influenced the adsorption equilibrium [16]. Cation exchange was the main mechanism of soil adsorption of heavy metals. In general, more H^+^ would compete with the adsorption site of Fe^3+^ in the solution at a lower pH value, resulting in a lower adsorption capacity of Fe^3+^ under the condition of low pH. Therefore, comparing these initial pH value, the pH value of 4.20 was more conducive to the adsorption process of soil.

### 3.3. Analysis of Adsorption Kinetics

**Figure 3:**
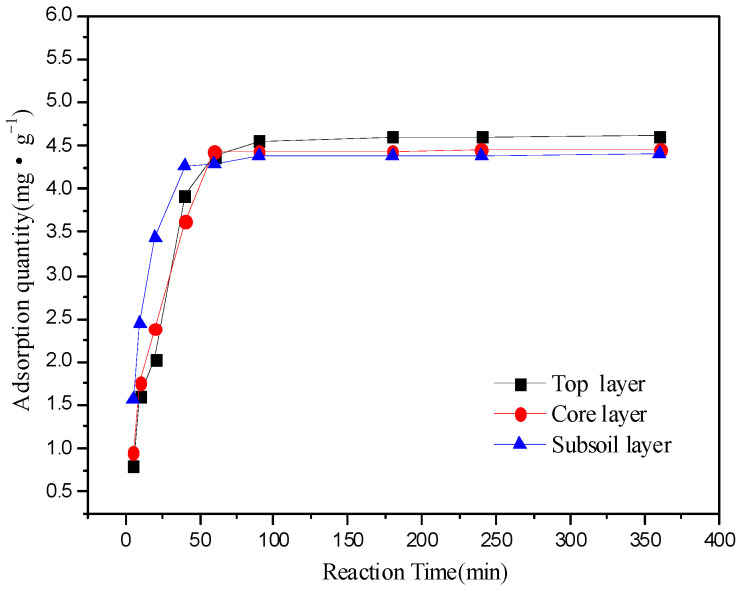
Adsorption kinetic characteristics of each soil. (4 mg of soil, initial concentration of Fe^3+^ was 300 mg·L^−1^ and the initial pH was 4.20, 25 °C).

The adsorption kinetics of the three layers of soil (top layer, core layer and subsoil layer) were analyzed under conditions where the initial concentration of Fe^3+^ was 300 mg·L^−1^ and the initial pH was 4.20. As shown in the Figure 3, the adsorption of Fe^3+^ in soil could be divided into two stages of fast and slow, which was a common phenomenon of the adsorption reaction. Experimental data were fitted with the first-order kinetic equation, second-order kinetic equation and Elovich equation, and the fitting results were shown in Table 2.

The fitted correlation coefficients R^2^ of the kinetic equation were shown in Table 2 and showed that the adsorption of Fe^3+^ on each layer of soil was correlated with the three kinetic equations. Moreover, the adsorption of Fe^3+^ on soil was most consistent with the second-order kinetic equation. The correlation coefficient R^2^ of second-order kinetic equation was close to one, and the theoretical value of maximum adsorption capacity was very close to the measured value. For the first-order kinetic equation, the correlation coefficient R^2^ was between 0.82 and 0.99. And for the Elovich equation, correlation coefficient R^2^ was between 0.87 and 0.93. These results indicated that the adsorption of iron ions in the three soils was mainly by chemical adsorption, and chemical adsorption was the main step to control the adsorption rate.

**Figure 4:**
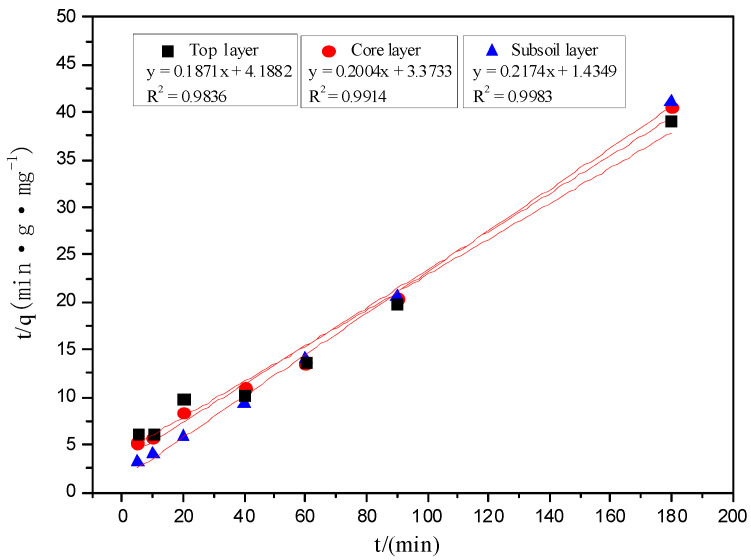
Second-order kinetic equation fitting curve.

The second-order kinetic fitting curve was shown in Figure 4. The second-order kinetic adsorption constant for the subsoil layer was about two and three times of that in the core layer and the top layer, respectively. Meanwhile, the adsorption rate of the subsoil layer was significantly faster than that of the other two layers, which was consistent with the results of Section 3.1 and Section 3.2. Some research reported that appropriately increasing organic matter content in soil could enrich the chemical groups and promote the adsorption effect of heavy metals [17,18]. According to the results in Table 1, the organic matter content and CEC of the subsoil layer were higher than those of the other two layers, so the subsoil layer showed better adsorption capacity.

### 3.4. Analysis of Isothermal Adsorption Kinetics

**Figure 5:**
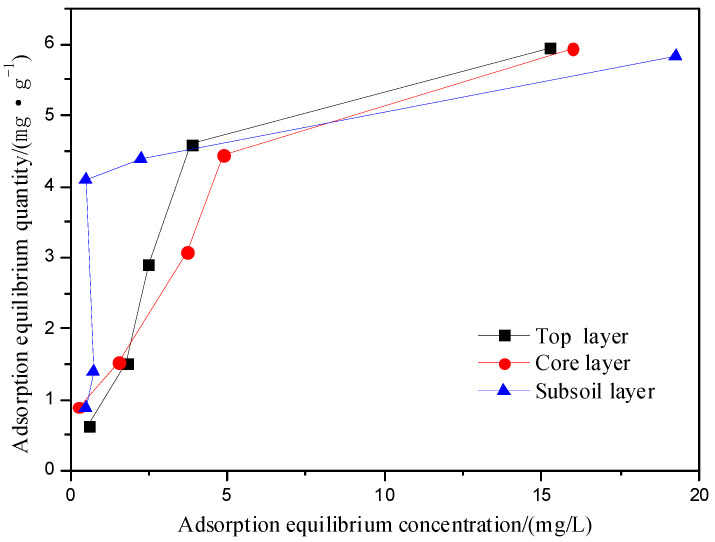
Adsorption Isothermal curve. (4 mg of soil, initial concentration of Fe^3+^ was 100, 200, 300, 400 mg·L^−1^ and the initial pH was 4.20, 25 °C).

The isotherm adsorption curves were shown in Figure 5. The results of fitting test data with the Freundlich model and the Langmuir model are shown in Table 3. The slope of isotherm adsorption curves of three soils decreased with the increase of the concentration of equilibrium solution and finally became flat, which was a typical L-shaped adsorption [19]. The reason may be that the metal at low concentration first combines with the high adsorption site of the soil and then gradually combines with the low adsorption site of the soil after the high adsorption site is gradually saturated, and finally the ions in the soil and solution gradually reach a dynamic equilibrium.

The Langmuir equation assumes that the adsorption is monolayer adsorption on a uniform surface, and the Freundlich equation assumes that the adsorption is multilayer adsorption on a heterogeneous surface [20].

According to Table 3, the Langmuir model can better fit the isothermal adsorption process of Fe^3+^ in the three soils, which indicates that the adsorption process is mainly a monolayer adsorption process. This is consistent with the research results of Xu et al. [21].

The fitting constant 1/*n* can reflect the adsorption strength [22]. In the Freundlich model, 1/*n* of the subsoil was between 0–1, indicating that the adsorption reaction of the subsoil to iron ions was the easiest to carry out [23].

### 3.5. FTIR Analysis of Soil before and after Adsorption

**Figure 6:**
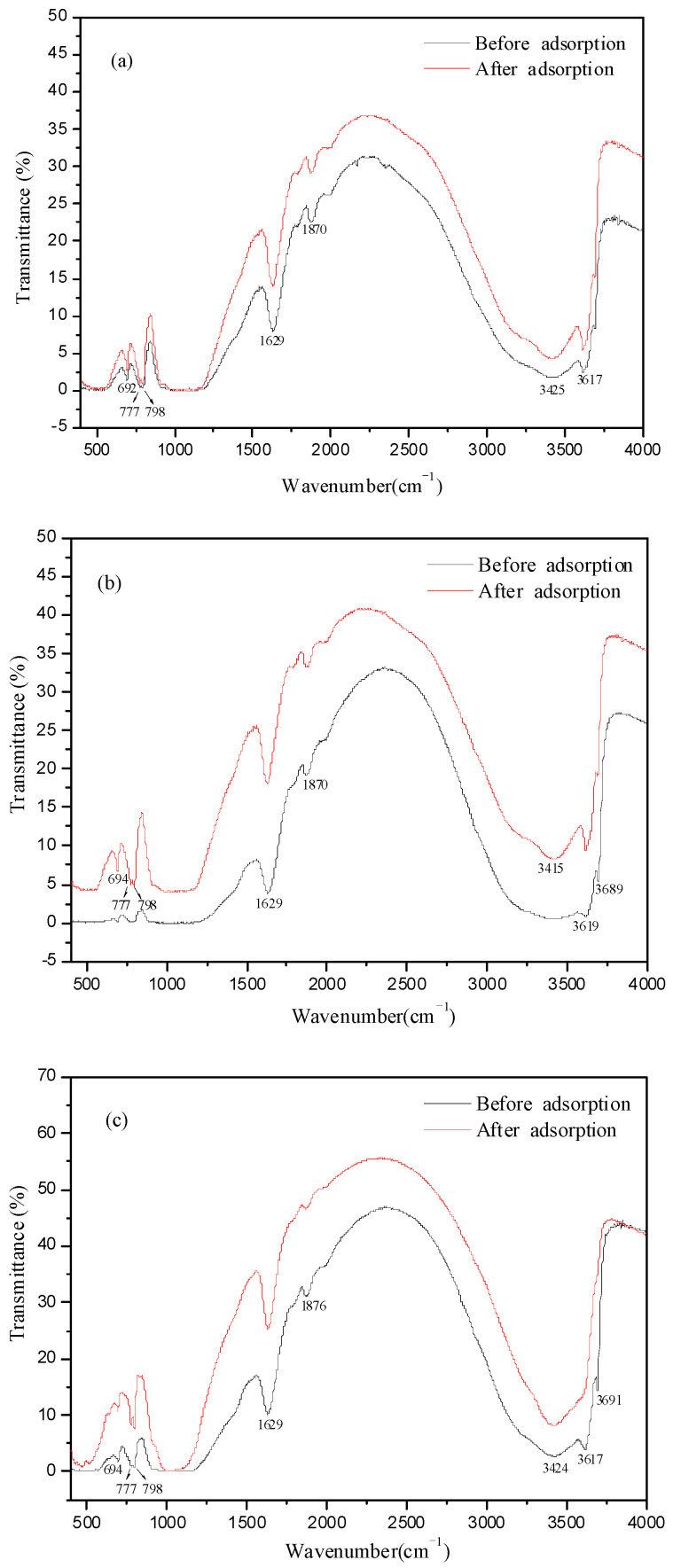
Infrared spectra of each soil before and after Fe^3+^ adsorption: (**a**) top layer of loess soils; (**b**) core layer of loess soils and (**c**) subsoil layer of loess soils.

The infrared spectra of the three soils before and after Fe^3+^ adsorption were shown in Figure 6. The three soils all belonged to yellow soil, so the characteristics of the infrared spectra were very similar. The wide absorption bands near 3430 cm^−1^ and 1028~1032 cm^−1^ were typical infrared spectrum absorption characteristic peaks of 2:1 clay minerals, indicating that there was a large amount of montmorillonite in the soil [24,25]. The absorption band at 694 cm^−1^ was a typical infrared spectrum absorption characteristic peak of kaolinite. The infrared spectrum of yellow brown soil with quartz double peaks of 798 cm^−1^ and 778 cm^−1^ were ascribed to the Si-O group, and the characteristic peak at 3700~3100 cm^−1^ was ascribed to the -OH stretching vibration band. The peak at 1630 cm^−1^ belonged to the C=C group and the peak at 1870 cm^−1^ was belonged to the C=O group, which were characteristic peaks of organic components in soil. The above functional groups in soil provided favorable conditions for the adsorption of Fe^3+^ through chemical adsorption. The absorption peaks at 798 cm^−1^ and 778 cm^−1^ in the core and subsoil layers were significantly enhanced, indicating the adsorption between the Si-O group and Fe^3+^ [26,27]. The C=O and -OH groups in the subsoil layer were weakened and disappeared obviously after the adsorption, indicating that the chemical adsorption in the subsoil layer was very obvious, and the adsorption performance was better than that in the top and core layer.

## 4. Conclusions

The adsorption capacity of Fe^3+^ in each soil layer was increased gradually with the increase of initial concentration and reaction time. The maximum adsorption capacity of Fe^3+^ in these three soils was about 6 mg·g^−1^ when the initial concentration of Fe^3+^ was 400 mg·L^−1^. The adsorption rate of subsoil layer at pH 4.20 was faster than that at pH 3.06, but it showed little effect on the adsorption rate of top and core soil. More H^+^ ions would compete with the adsorption site of Fe^3+^ in the solution at a lower pH value, resulting in a lower adsorption capacity of Fe^3+^ under the condition of low pH. Therefore, the pH value of 4.20 was more conducive to the adsorption process of soil. Moreover, the adsorption of Fe^3+^ on soil was most consistent with the second-order kinetic equation. The correlation coefficient R^2^ of second-order kinetic equation was close to one and the theoretical value of maximum adsorption capacity was very close to the measured value. The Langmuir model can better fit the isothermal adsorption process. These results indicated that the adsorption of iron ions in the three soils was mainly by chemical adsorption, and it was the main step to control the adsorption rate. The organic matter content of the subsoil layer was high, and it contained rich functional groups such as C=O and -OH groups. The infrared spectrum results showed that the peaks of the subsoil layer were weakened and disappeared obviously after the adsorption, and the chemical adsorption effect was obvious. Functional groups in soil provided favorable conditions for the adsorption of Fe^3+^ through chemical adsorption. The C=O and -OH groups in the subsoil layer were weakened and disappeared obviously after the adsorption, indicating that the chemical adsorption in the subsoil layer was very obvious.

## Figures and Tables

**Table 1:** The physical and chemical properties of each soil.

Soil Sample	pH	Organic Matter (%)	CEC (cmol·kg^−1^)	Background Value of Iron (mg·kg^−1^)	Background Value of Manganese (mg·kg^−1^)	Granulometric Composition (%)		
						Clay	Particle	Sand
Top layer	5.88	5.7	12.16	38.21	2.52	35.534	33.542	30.924
Core layer	3.86	4.6	9.97	38.6	8.87	37.439	32.769	29.792
Subsoil layer	6.24	6.14	13.47	42.79	23.76	39.646	31.690	28.664

**Table 2:** Adsorption kinetic parameters.

Soil	Elovich Equation *q* = *b* + *k*ln*t*			First-Order Kinetic Equation ln(*q_m_* − *q*) = ln*q_m_* − *kt*				Second-Order Kinetic Equation *t*/*q* = 1/*k q_m_*^2^ + *t*/*q_m_*			
	*b*	*k*	R^2^	*k*	*q_m_* (mg·g^−1^)	*Q_m_* (mg·g^−1^)	R^2^	*k*	*q_m_* (mg·g^−1^)	*Q_m_* (mg·g^−1^)	R^2^
Top layer,	−1.1147	1.2185	0.9196	0.0538	5.6644	4.5981	0.9931	0.01129	5.3447	4.5981	0.9836
Core layer	−0.7135	1.1079	0.9288	0.0839	6.7524	4.4406	0.8261	0.01503	4.9900	4.4406	0.9914
Subsoil layer	0.6375	0.8327	0.8725	0.0729	3.9484	4.3838	0.9718	0.03627	4.5998	4.3838	0.9983

*q_m_* was the theoretical value of the equilibrium adsorption capacity; *Q_m_* was the measured value.

**Table 3:** Adsorption Isothermal Parameters.

Soil	Langmuir Equation *C_e_*/*q_e_* = *C_e_*/*q_m_* + 1/(*b* × *q_m_*)			Freundlich Equation ln*q_e_* = ln*K* + (1/*n*) × ln*C_e_*		
	*q_m_*	*b*	R^2^	*K*	1/*n*	R^2^
Top layer	0.4138	0.5519	0.8885	10.0232	1.5761	0.4722
Core layer	0.5230	5.1787	0.9142	0.2437	1.7919	0.489
Subsoil layer	6.3980	0.5543	0.9798	2.2739	0.3555	0.4468
